# "The Hospital" Nursing Mirror

**Published:** 1898-12-10

**Authors:** 


"The Hospital" Nursing Mirror.

Being the Nursing Section of "The Hospital."

[Contributions for this Section of "The Hospital" should be addressed to the Editor, The Hospital, 28 & 29, Southampton Street, Strand, London, W.C., and should have the word "Nursing" plainly written in left-hand top corner of the envelope.]

### News from the Nursing World.

### THE QUEEN AND HER WOUNDED SOLDIERS.

Once more, last Saturday, the Queen has made a pilgrimage to the Military Hospital, Netley, to see her wounded soldiers. She arrived shortly after two o'clock from Windsor, and drove at once to her destination. Princess Henry of Battenberg and the Princess Victoria of Schleswig-Holstein accompanied her, and Lord Kitchener of Khartoum was amongst those that welcomed her. Seated in her wheel-chair, Her Majesty passed through the ranks of those sufficiently well to be mustered in the corridor, and was taken up in the lift, visiting first the surgical and then the medical wards, going to the bedsides of the more seriously ill. By her especial request the proceedings were quiet and private, and representatives of the Press were excluded. In all this the Queen has acted with excellent taste. In addition to the pleasure of receiving their Sovereign's thanks in person, 147 men wounded at Omdurman and Atbara were decorated with medals commemorating these events by Lord Kitchener.

### THE PRINCESS LOUISE AND COOKERY.

Everyone knows that the members of the Royal family are adepts in housewifely arts, and that when children they had a delightful kitchen fitted up for their special edification and pleasure. Indeed, there is a story vouchsafed as true to the effect that the Princess Royal was happily engaged in some culinary mystery when called upon to sign her marriage contract, and that she did not approve of the interruption. Be that as it may, however, no one in the world recognises more truly the vast importance of good cooking, both in health and sickness, than the ladies of our Royal House. The visit, therefore, of the Marquis and Marchioness of Lorne to the Edinburgh School of Cookery is quite in accordance with the spirit they have always shown towards every effort made in the interests of domestic comfort. The Marquis's few words, on behalf of his wife, were highly significant of her attitude towards the action of the school inspectors in discouraging instruction in cookery being given in schools. He emphasised the fact that, if cooking were not taught in schools, instruction in the subject would have to be given by establishing institutions more or less separate from them, for to cook somehow or other the people must be taught. With such a dictum who can disagree? Certainly not doctors and nurses.

### LADY DUFFERIN ON LONDON NURSES AND NURSING.

Lady Dufferin presided at the annual meeting of the Belfast Nurses' Home, which was held at the house in Frederick Street on November 30th, 1898. Many influential subscribers attended, and the society shows not only growth but an appreciation for the higher requirements, both of nursing and nurses, that is eminently satisfactory. Until the present year the late Lady Shaftesbury has taken the chair at the annual meetings, and many sincere regrets were expressed at her loss. In Lady Dufferin, however, the association has found a successor in whose hands its best interest will certainly not be less efficiently served. Her ladyship, it may be remembered, identified herself with nursing work in India during her husband's viceroyalty. She has since her return inspected the methods of hospitals and nursing homes in London, under the guidance of the chairman of the London Hospital, whose axioms on nursing matters she quoted in her address, adding thereto her own opinion: "I think," she said, "no one can walk through a London hospital without remarking upon the nurses; they are known everywhere as the best trained nurses in England, and not only is their professional training attended to and their comfort, but also, I may say, their manners and their conduct, and everything that makes a good nurse. I hope, and feel sure, that here we are trying to keep up with the times; that our Nursing Home will keep improving, and will be as good as the new hospital we are going to have, and that every effort will be made and every possible care taken to improve and perfect the nurses." The new hospital here alluded to is the Victoria Hospital, which is expected to be fully equipped in the course of two or three years.

### AN INTERESTING MARRIAGE.

It will interest nurses to learn that a marriage is arranged between Mr. Lewis Vernon Harcourt, son of Sir William Harcourt, and Miss May Burns, daughter of the late Mr. Walter H. Burns, late chairman of the Royal National Pension Fund for Nurses. Miss Burns' mother, Mrs. Walter H. Burns, was the daughter of the late Junius S. Morgan, after whom the Benevolent Fund of the Pension Fund is named, in memory of his great generosity to the Pension Fund and to the Benevolent Fund, a generosity which has been continued in a munificent manner by his family. Already we have received proposals from nurses that a subscription should be started amongst members of the Pension Fund to present Miss Burns with a wedding gift. We have not consulted Miss Burns on the subject, but we feel sure we are expressing her feelings when while heartily endorsing the wish expressed to demonstrate a kindly feeling towards her, we suggest that this might well be done in the form of a short address of congratulation, nicely illuminated and framed. We shall be pleased to carry this proposal out, if it meets with the wishes of the nurses; and if we receive not less than 200 letters expressing a wish to join in this graceful act expressing kindly feeling and enclosing not more than six stamps. The letters will be acknowledged in our columns, and a statement of accounts will be sent to each subscriber if the scheme is carried out. Will nurses desiring to join in the scheme address their letters to the Editor, The Lodge, Porchester Square, W., with the word "Congratulation" in one corner to obviate trouble? The names of the contributors would, of course, be appended to the congratulatory address.

### AT THE ORTHOPÆDIC HOSPITAL.

The annual sale of work at the Orthopædic Hospital, Great Portland Street, is often very enjoyable, and that held on the 2nd and 3rd inst. was no exception to the rule. There was such a friendly undercurrent, sellers and buyers were so cheerful and busy that no one could be surprised to hear that the handsome new lockers, the invalid chair, and even the support of a free cot have all been provided from the "Extra Comfort Fund," for which these sales were organised. The Duchess of Marlborough was unfortunately unable to attend. Her Grace is generally present at anything of the kind, as she takes great interest in the hospital, and her husband is the president. The out-patient department looked animated and pretty. The stalls which were held by the members of the Ladies' Committee were gay with art needlework, pictures, and nick-nacks, with many a humbler bit of sewing tucked in between, and an additional attraction was offered this year by a couple of lectures each day from Mrs. Archibald Little, who founded the Natural Foot Society in China. The one was on "Foot Binding," and the other on "The Position of Women in China." Mrs. Little has travelled in China with her husband, and very graphically described things as she saw them. Part of the programme on these occasions is to visit the children's ward, and there to see the plump, merry children at different stages on their way to recovery. A bonny wee man, somewhat sorrowful at his new surroundings, was the latest inmate. His soft, white little foot was seen to be drawn up to one side, a state of affairs which, if neglected, would cripple him for life. Now, however, in a few months the little fellow will be well.

### OUR CLOTHING DISTRIBUTION.

Whilst gratefully acknowledging two more parcels of clothing for our distribution, from Miss Lucy Eastcoll and "M. H." (Ayr) respectively, we beg to remind our friends and helpers in this good work that we are anxious to have our contribution to the Christmas happiness of the convalescents in hospital ready in good time this year. One pound from the "Mirror," 5s. and 2s. 6d. from other contributors, have been received, which will be expended in the course of the week. Our readers will materially help if they will send in their gifts as soon as possible. Whosover giveth quickly giveth twice.

### ABERDEEN ROYAL HOSPITAL FOR SICK CHILDREN.

A bazaar in aid of the funds of this hospital was held lately in Aberdeen, and was opened by H.R.H. Princess Henry of Battenberg. Her Royal Highness was received at the station by the Lord Provost and Magistrates of the city and by the directors of the hospital, and at the entrance to the bazaar by Miss Lumsden, Hon. Superintendent of the hospital, and by the hospital staff. Her Royal Highness made many purchases at the various stalls, and expressed much sympathy with the object of the bazaar, viz., to liquidate the debt on the new Nurses' Home. The sum realised by the two days' sale was over £1,430. The new home, which adjoins the hospital, contains dining-room, sitting-room, and bed-room; accommodation for thirty nurses also a sick-room. The home is comfortably but very plainly furnished, all unnecessary expense having been rigidly avoided. Besides this very needful addition to the hospital, a further extension is contemplated as soon as the funds permit, there being still urgent need of an enlargement of the wards and of a new and suitably-equipped theatre. Yet, in spite of many difficulties, good work is being done by the hospital. This year over 840 patients have been nursed in its wards, and 380 surgical operations have been performed. Upwards of 70 private cases have been attended by the nurses, who are in great request, especially for cases of diphtheria and tracheotomy.

### AFFAIRS AT RANGOON.

It is very pleasant to be able to state definitely that the recent disturbance at Rangoon had nothing whatever to do with our English nurses. The five nurses who resigned are Eurasians on the staff of the Municipal Hospital. Sister Ida Gould, who had been engaged in leper work, was appointed by a civil surgeon to the plague reserve staff not very long ago, and was thence promoted to the position of matron of the permanent staff. She seems not to have been at all popular, and the five nurses referred to left the hospital before the expiration of their due notice. The two nurses from St. George's are also on the plague reserve, and in the absence of that most undesirable visitant have been proving their worth in the wards. Eleven typhoid patients have been sent there from the s.s. "Cheshire," who are reported to have contracted the disease in consequence of an impure ice supply, and the additional skilled nurses have been most welcome.

### SHORT ITEMS.

The Elgin District Nursing Association's third report shows a favourable state of affairs. Small subscribers are increasing, the credit balance is higher, and two nurses are at work; whilst the medical men are availing themselves of the advantages they offer.—The Russian Red Cross Society, like that of America, occupies itself in times of peace in assuaging the suffering caused by internal calamities. The Czar has recently subscribed half a million roubles to its coffers for the relief of the peasantry distressed by the failure of the harvest.—£110 was raised last week for the Sick Nursing Fund of St. Matthew's, Exeter, by a two days' sale. — A monthly entertainment has been arranged at the Hospital for Paralysis and Nervous Diseases, Regent's Park, N.W., by Miss Lamport and Miss Gethen, by the permission and co-operation of the matron. The entertainments are varied in character, and sometimes the music is excellent. A point is made of giving no trouble to the authorities.—Lady Grey has sent the Governors of Hartlepool Hospital £700 to provide the nursing accommodation which is wanted so much.—The Queen Victoria Nurses' Institute, Cardiff, is to have the earnings of some amateur theatricals which took place last week at the Grand Theatre. "Harmony" and "Our Regiment" were the pieces selected for representation, and were duly appreciated by a crowded house.—The Nurses' Home of the Gillingham Jubilee Nursing Institute is now complete. It is situated in Queen Street, and a water supply has been arranged from a neighbour's well.

### Nursing in Diseases of the Throat, Nose, and Ear.

By MACLEOD YEARSLEY, F.R.C.S.Eng., Assistant Surgeon to the Royal Ear Hospital, Surgeon-in-Charge of the Department for Diseases of the Throat, Nose, and Ear, the Farringdon General Dispensary, Hon. Surgeon for Diseases of the Throat and Ear, the Governesses' Home, &c.

### IX.—AFTER - TREATMENT OF OPERATIONS ON THE THROAT.

THE after-treatment of cases in which the galvano-cautery has been applied to granular pharyngitis is simple. The patient should take only bland, cold food for a day or two, and avoid smoking. If any extensive burning has been done, and much discomfort is complained of, the sucking of ice will give much relief, or a cocain pastille may be used occasionally. A simple saline or cleansing spray may be used for a few days after the cauterisation, which is generally healed in a week or less. These remarks apply also to cauterisation of the lingual tonsil.

After uvulectomy, soft, bland, and cold food should be taken and a cocain lozenge sucked before meals to minimise or abolish any pain on swallowing. Very occasionally removal of the uvula is followed by secondary hæmorrhage. Should this occur, it does so about three days after the operation. A styptic gargle should be used in such cases, one of tannic and gallic acids being very useful.

The after treatment of removal of the tonsils is one of common sense. Much of what has already been said of the after treatment of adenoids (Article VII.) applies here. It is certainly wiser to confine the patient to the house for a day or two after the operation, and to keep him in a warm and equable temperature. In doing so, however, the necessity for pure air should not be lost sight of, for when a patient is ordered to be kept in a warm room, those who have charge of him too often seem to consider that the order means confinement to a hot, stuffy room, in which ventilation and fresh air are conspicuous by their absence.

For the first few days the food should be of a soft, bland character. A mild antiseptic gargle or spray should be used, and to minimise the pain of swallowing, a cocain lozenge or pastille may be sucked before taking food. Pain may be relieved by sucking ice.

Much has been said or written as to undue hæmorrhage after tonsillectomy. Dangerous hæmorrhage is rare if proper precautions have been observed. According to one writer, who has reviewed the literature upon the subject, the causes of hæmorrhage after removal of hypertrophied tonsils are: (1) Abnormal distribution of the blood vessels of the tonsils; (2) hæmophilia; (3) over-use of the voice; (4) the too early eating of solids; (5) the recorded cases of severe hæmorrhage have occurred very frequently after the use of the bistoury. When severe after-hæmorrhage occurs, it is nearly always in adults. It will be noted that the too early eating of solids is given as a cause of this complication; the nurse who has charge either of cases of removal of tonsils or of an out-patient department where that operation is often performed, will do wisely to impress upon the patients the necessity of adhering to a soft diet for several days after the tonsils have been removed.

Should the nurse find herself with a case in which severe hemorrhage has occurred after tonsillectomy, she should, if it be very copious or does not quickly cease, send at once for the surgeon. Should the bleeding be slight, quiet and ice to suck may be sufficient to stop it. But if it be persistent, a mixture of one part of gallic acid to three parts of tannic acid dissolved in water may be applied by means of a swab to the bleeding parts, or the same powder may be made into a paste and rubbed on to the tonsil with the finger.

Should, however, the bleeding be copious and sudden, the nurse requires to act promptly. If she understands how to compress the common carotid artery she should do so, or she should open the patient's mouth, thrust her finger on to the bleeding part, and press outwards against her thumb placed outside behind the angle of the jaw. She can thus at least control the hemorrhage until the arrival of the surgeon, who may require to seek for, seize and twist with torsion forceps the bleeding point, or touch it with the cautery; failing which it has in some cases been necessary to proceed to ligation of one of the carotid arteries.

After the incision of a peritonsillar abscess the treatment will be much the same as that already described for other operations about the tonsil. Frequent gargling with a warm antiseptic solution should be practised, together with cocain pastilles, soft, unirritating foods, &c.

Passing now to the after-treatment of the more serious operations upon the larynx, the nurse will in all probability have gained some experience therein whilst training at a general hospital. Foreign bodies in the larynx may be dismissed at once; if an operation such as subhyoid pharyngotomy, or tracheotomy, has been required for their removal the after treatment of those operations should be consulted. After removal of a foreign body by the natural passages some care is of course necessary, and the surgeon should be consulted as to his wishes in the matter.

As regards intubation there are certain accidents to be guarded against. The larynx and trachea soon become tolerant to the tube, and the urgent dyspnœa for which the intubation was performed being relieved, the patient usually drops into a comfortable sleep. As a matter of fact, the intubation tube is worn much more comfortably than a tracheotomy tube and when *in situ* is often not felt at all by the patient. The accidents to be guarded against are as follows: The tube may be coughed out, with an immediate return of the dyspnœa. This is a rare occurrence, but should the nurse experience it she should send for the surgeon without delay, and make no attempt to reinsert the tube. The tube may become blocked by false membrane, an accident that can only occur in a very feeble patient, as the tube is always expelled by a vigorous cough when it becomes blocked.

The after-treatment of cases of radical operations on the larynx, especially of those performed for malignant disease of that organ, was up to a recent period exceedingly troublesome and difficult to both patient and operator, and requires undivided and unremitting care and attention on the part of the nurse.* The former method was to leave the tampon cannula *in situ* for twenty-four hours after the operation, and to then substitute an ordinary tracheotomy tube covered with indiarubber for several days up to one week. The interior of the larynx was plugged twice daily with iodoform gauze up to the fifth or sixth day, when this plugging was only done once a day. For the first three or four days feeding was effected by means of a soft œsophageal feeding-tube. The comfort of the patient was conspicuous by its absence, the plugging with iodoform gauze giving rise to much irritation with violent and incessant coughing. By the introduction of Mr. Butlin's method of procedure† the great discomforts to which patients were formerly subjected have been greatly modified. The tampon tube is now removed at once, and no tracheotomy tube substituted. This is decidedly one of the most important changes, as it was almost impossible to keep the tampon-cannula aseptic, a fact which seriously contributed to the occurrence later of

* Semon. "The Results of Radical Operation for Malignant Disease of the Larynx, from Experiences of Private Practice."—*The Lancet*, Dec. 29, 1894, p. 1,533.

† *Vide* "Proceedings of the Laryngological Society of London," Oct. 11, 1893, p. 28.

septic complications. The patient is no longer propped up in bed, but lies quite horizontally, with one small pillow only under his head. He lies on the side corresponding with the half of the larynx which has been the seat of operation, in order to allow the secretions from the wound to run out directly into a small pad of iodoform gauze. The wound is covered with a bandage of similar material. The nurse renews this bandage of iodoform gauze as often as it becomes saturated with secretion. The wound is also frequently dusted with a mixture of iodoform and boric acid.

The question of feeding is, of course, one of very great importance. As a rule, even on the first day, the patient will be able to take a little liquid nourishment by the mouth. The precaution should, however, first be taken of giving him a little water through a suitable feeder, and, should it come through the wound, he must be fed by nutrient enemata or by a stomach pump. Sir Felix Semon points out that a very good plan is to let the patient lie sideways with his body partly over the side of the bed, head downwards, and let him drink from a feeder introduced into the lower-lying side of his mouth; by this means the fluid that he drinks completely avoids the larynx, and thus no cough is excited.

The great risks of these radical operations upon the larynx lie in the occurrence of bronchitis, pneumonia, and other septic sequelæ; these usually occur in the course of the first week. They are mentioned here because their avoidance not only depends upon the strictest antiseptic and other precautions during the operation, but upon the unceasing attention which the patient receives during the first few days of convalescence. In uncomplicated cases it has happened that patients have been able to get up on the fourth day after thyrotomy, and leave the house on the twelfth day. It is to the improvements which have been introduced of recent years into both the method of operation and the after-treatment that the rate of mortality of these radical operations has so much improved.

### Training in the Provinces.

(*Continued from page* 94.)

### ROYAL BERKSHIRE HOSPITAL, READING (160 Beds).

### Terms of Training.

A THREE years' course of training is offered to nurses at the Royal Berkshire Hospital. Candidates should be between 23 and 35 years of age, and are received in the first instance on a month's trial, after which, if accepted, they enter into a four years' engagement with the hospital. No premium is required from three years' probationers, salary commencing the first year at £10, rising to £15 the second, and to £18 the third year. Staff nurses' salaries range from £24 to £34 per annum, and the hospital pays half premium up to £7 a year towards the Royal National Pension Fund in addition. Laundry and indoor uniform are provided for all the staff, and outdoor uniform is also given to the private nursing staff. In their third year nurses are able to gain experience in private nursing, being sent out to assist the nurses employed on the private staff. Lectures are given to nurses during their training by members of the visiting staff on anatomy, physiology, hygiene, nursing, &c., and certificates are granted to those who satisfactorily complete their three years' engagement. Night duty is taken by third year and staff nurses, for three months at a time, alternately with day duty. For promotion to the charge of wards, three years' training is an essential qualification.

A certain number of lady pupils are received for one year's training, paying a premium of 24 guineas. They are on precisely the same terms as the ordinary probationers with regard to work and treatment, and are provided with indoor uniform and a certain amount of laundry.

### Hours On and Off Duty.

Probationers' hours on duty are between 7 a.m. and 9 p.m., with two hours off duty daily, and time for meals. Sisters go on duty an hour later in the morning. Days off are given when convenient, and on these days nurses do not enter the wards at all. Night nurses are on duty from 9 a.m. to 9 p.m. Three weeks' annual leave is given.

### Meals.

All meals are served in the nurses' dining-room, and no "food allowances" are made to the nurses. They are supplied at each meal with as much as they require, and care is taken that the food is varied and well cooked. The hours of meals for the day staff are as follows: Breakfast, 6.30 a.m. for the nurses, 7.30 for sisters; half-an-hour for luncheon between 9 and 11; dinners, 1.30 and 2 p.m.; tea, 5 to 6, for which half-an-hour is allowed; and supper, 8.30 to 9, or 9 to 9.30.

### SALFORD ROYAL HOSPITAL (125 Beds).

### Terms of Training.

APPLICANTS for training at the Royal Salford Hospital, Manchester, should be between 20 and 35 years of age. If accepted after one month's trial, a three years' course of training is entered upon, a certificate being granted at its satisfactory conclusion, and after passing examination. No premium is required, salary beginning at £6 the first year, and rising to £15 the second and to £20 the third year, and after four years' service to £25. Sisters' salaries range from £25 to £31, by a yearly increase of £2. Laundry, in and out-door uniform are provided by the hospital. Lectures are given by members of the visiting staff on physiology, anatomy, and medical and surgical nursing. During their third year probationers, if considered competent, may be sent to private cases. The full term of three years' training is a necessary qualification for promotion to the charge of wards. Night duty is taken by assistant nurses, probationers in their second and third years, for periods of two months in alternation with day duty.

### Hours On and Off Duty.

The day staff are on duty between the hours of 7 a.m. and 9 p.m.; from this time must be deducted 4½ hours for recreation and meals, daily, bringing the working hours down to 9½.

Extra time off is given every other Sunday, a half-day weekly, and week ends when they can be arranged for.

Night nurses are on duty from 9 p.m. to 9 a.m.

A fortnight's holiday is allowed in the year.

### Meals.

All meals are served in the dining-room, and no food allowances are made to the nurses except to those on night duty for their midnight meal. Day nurses breakfast at 8.30 a.m.; their dinner is at 12.30; tea at 4.30; and supper at 9 p.m.

### The Convalescent Fund.

WE have much pleasure in acknowledging the receipt of 10s. to our Convalescent Fund from Miss Katherine Tilt. We shall be glad if others will follow her good example, as our treasury is very low just now, and applications for our help are more frequent.

### On Nursing in Switzerland.

THE FOUNDATION OF THE FIRST SWISS TRAINING SCHOOL FOR NURSES.

MOST likely the readers of this journal will be astonished to hear that Switzerland, a country which we may proudly say stands high in matters concerning education, does not possess a training school for nurses. For a long time in Germany, and even more so in England, such schools have been developed, and are highly appreciated by the population. The cause of our being so far behind other nations in this matter is that nursing with us has up to now been practised chiefly by members of Protestant and Roman Catholic religious orders. There has also been founded a hospital by the Verein für freies Christenthun, which serves as a training school for Red Cross sisters. Free nursing as a profession was regarded by the public opinion as a rather subordinate occupation, and consequently mostly girls of comparatively little education devoted themselves to it. Many young women who not only felt inclined and talented for the vocation, but even such as were possessed by an ardent desire for nursing work, could not devote themselves to it unless they were prepared to give up their personal freedom and by pledging themselves to an order. If such a young woman was obliged not only to earn her own livelihood, but also to be an aid to her family, she could not enter an order, and to become a mere hired nurse was objectionable for the reason stated above. At the same time, the hospitals and other public institutions, as well as private individuals, require well-trained nurses, for the diaconal associations cannot comply with all demands for sick nursing. Hence there exists in Switzerland a strong desire for nursing work, as well as a widespread demand for good nurses, and to solve the problem the "Schweizerische Gemeinmitzige frauenverein" (an association of women of all ranks for humanitarian work) resolved 18 months ago to take in hand the reorganisation of nursing in the country. The object is to elevate the profession of nursing the sick to a position which will no longer be unfairly undervalued, but which will enjoy the general esteem due to the noble work. It will no longer be bound to rules of religious orders, and it will procure for those practising it a competency in old age. The above-named association formed a special commission for the purpose of finding a solution of the matter. The commission consists of several lady doctors, nurses, and a few other members, and Miss Anna Heer, doctor of medicine in Zurich, is president. The commission resolved, in the first instance, on the foundation of a Swiss school for the training of nurses, and at the same time the organisation of a union among the nurses themselves. For practical instruction a hospital will be required, suitable in its arrangements and organisation for the purpose in view. As the existing infirmaries and hospitals are partly unsuitable and partly inaccessible for training purposes, we shall be obliged to build our own hospital, which is to be in Zurich. It will receive only female patients, and the training of the nurses will be under the direction of the lady doctors.

The following are the provisional rules of the training school, viz.:—

1. The training school of the Swiss "Gemeinmitzige frauenverein" is a Swiss institution, and stands on a humanitarian ground. Its objects are: (*a*) The training of women and girls as general or monthly nurses for practice in private circles, in hospitals, or as district nurses; (*b*) the training of ambulance nurses for the Geneva Cross (Geneva Convention); (*c*) the organisation of repetitional courses of study; (*d*) the formation of a Swiss society of free, sick, and monthly nurses, with insurance against sickness and old age.

2. The age for admission will be 18 to 35 years.

3. The admission to take place every year in spring (May 1st). A written application is to be presented to the superintendent at least one month before the training course begins. To the application must be added: (*a*) A short statement of the candidate's previous life and education, composed and written by herself; (*b*) a certificate of birth; (*c*) an official attestation of character; (*d*) school certificates; (*e*) in the case of minors, the written consent of the father or guardian.

GENERAL NURSES.

4. The training course for nurses will occupy three years, and will be practised at the hospital connected with the school and in the public hospitals.

5. The school training will occupy one year, and pupils will be instructed theoretically and practically.

6. Theoretical instruction will be given in the elements of anatomy, nursing of both the healthy and the sick, and in ambulance service.

7. Practical instruction will be given in the duties of the sick bed, in the sick-room, in the surgical operation-room, in the preparation of medical prescriptions, and the first aid in accidents. The pupils will also be taught cooking for invalids, and bookkeeping. There will also be offered to them an opportunity for learning one of the other languages spoken in Switzerland (French or Italian). Pupils as well as patients will be left entirely free in the matter of religion.

8. The instruction will be gratuitous, but as a guarantee that they will remain at the school for three years, each pupil will pay 300 fr. (about £12), which will be returned at the end of the course, with interest. The guarantee may be given in the form of a bond. For poor pupils the guarantee may be raised by the scholarship fund. If a pupil leaves the school during the first year a sum of 40 fr. (£1 12s.) for every month's training will have to be paid as compensation. If the pupil leaves during the second or third year the compensation will be fixed in each case by the board of the school.

9. During the two years of external training, and after their examination, the nurses will receive a monthly salary of from 16s. to 20s.

10. Special rules for admission, training, salary, service, retirement, and dismissal will be fixed by regulations of the Board.

MONTHLY NURSES.

11. The monthly nurses also will be taught during one year at the school, and for the two following years they will nurse under the control of the School Board, and will be paid accordingly.

12. The theoretical and practical instruction of the monthly nurses will in the main points be the same as that of the sick nurses, with special reference to nursing of mother and infant.

13. Admission, retirement, and dismissal will be fixed as stated in paragraph 8, but for monthly nurses the guarantee will be for £8 only.

14. Instruction in ambulance work will be given according to the demand in courses of three months, under rules 6 and 7, and practical exercise will be afforded at the infirmary or by clinical cases. These courses may be attended by married ladies and girls as occasional assistants.

15. From time to time other shorter courses will be arranged with special regard to those who were trained elsewhere and wish to join the Swiss Nursing Association.

We regret to say the time has not yet arrived for these rules to be put in actual practice. The necessary means—about 500,000 fr. (£20,000)—which are required for the school and hospital are to be raised entirely by public benevolence. Our county is small, and we have very few sick people. so the means are coming in very slowly. An appeal to the public has been made throughout Switzerland for this work, and as a result 130,000 fr. (£5,200) have already been collected, a sum which gives hope that the building may be erected next year, and the school opened in 1900.

### The Colonial Nursing Association.

At the request of the Princess Henry of Battenberg the half-yearly meeting of the Colonial Nursing Association was held in the Imperial Institute on the 1st inst. Her Royal Highness was present. Lord Lock presided, and Mrs. Piggott, the founder and hon. secretary, read the report. Mrs. Joseph Chamberlain was also there. She is interested in the work, as is her husband (the Secretary of State for the Colonies). Sir C. J. Brown, Miss Kingsley (of West African fame), Sir Henry Burdett, Sir Charles Mitchell (Governor of the Straits Settlements), all spoke words in season either as to the value of the work, the greatness of the need the association supplied, or as to the initial and inherent difficulties of the task and the success with which these have been grappled and overthrown.

Mrs. Piggott occupied the first part of her report with a *résumé* of the origin and aims of the society. The origin was the awful waste of valuable young lives caused by the lack of skilled nursing; the aim is to found and maintain a strong central committee through which the colonies could select, import, and support trained nurses. The result of eighteen months' work is that the association has now nine nurses at work, two in the Mauritius, one in Cyprus, one in Perak, one in Selangor, two in Ceylon, and two in Bangkok, Siam. In addition to these the Colonial Office has sent out seven matrons and eleven nurses at the recommendation of the association.

The success hitherto attending the progress of the association and the increasing demand for nurses has raised the question of providing for its widening liabilities, and Mrs. Piggott made a modest request for some £150 a year more for the purpose. Most of the colonies, in spite of a little uncertainty as to the possibility of such a thing in some cases, have been self-supporting, and refunded the money granted towards the initial expenses by the parent committee, but the same need of nurses is even more greatly felt in poorer colonies, which can only bear a portion of the cost, and the power of the association to satisfy this demand rests with the subscribers. The clerical work, which has hitherto been undertaken by volunteers, has grown to such an extent that a paid assistant secretary has been appointed. An important part of the scheme of colonial nursing is to prevail upon the Government to permit a nurse or a couple of visiting nurses to be lodged at the Government hospitals and under the supervision of the resident matron. In this advance Hong Kong leads the way, and two highly trained nurses are now at the service of the residents there.

Miss Kingsley, in telling something of the work of science and nursing in West Africa, said that in the Bight of Benin out of forty men that went in only one came out alive, and she gave a vivid picture of the need of "fighting King Death with scientific weapons."

Sir Henry Burdett thanked Mrs. Piggott for relieving him, a private individual and much occupied, of requests to provide nursing institutions and nurses in various quarters of the globe at a moment's notice, and instanced such an application from Shanghai, where he was asked to equip a home, and to supply a staff of nurses within a twelvemonth. This was done, and at the end of the time stated a hospital was added, the staff doubled, and the home enlarged. He bore witness to the discouragement attendant on the founder's efforts "to plant her flag," to the remarkable vigour of the young institution, as well as to the amount of work so cheaply accomplished. He promised to undertake to collect £10 of the £150 required in small sums if others present would do the same.

Mr. Jones, of Liverpool, upon whose steamers the colonists rely for sea trips and ice—both essential to health in that region—followed Sir Henry Burdett, and promised £50 of the sum asked for, with the request that it might be given to the needs of West Africa.

Sir Charles Mitchell urged caution, and pointed out one or two inherent defects common to humanity which had shipwrecked associations coming under his own observation. The first was that nurses marry (this elicited from the hon. secretary that there had only been one marriage in three years); the second that nurses require supervision; and, lastly, that they are apt to quarrel amongst themselves.

### The Princess Louise at Edinburgh.

The Princess Louise, accompanied by the Marquis of Lorne and Miss Alma Tadema, visited the Training Home of the Scottish branch of Queen Victoria's Jubilee Institute for Nurses on December 2nd. Dr. Joseph Bell, chairman of the committee, and Miss Guthrie Wright, the hon. secretary, received Her Royal Highness, who was presented with a bouquet. The Princess then gave the silver badge of the institute to Miss Cooper, assistant inspector of the Scottish District, in recognition of services rendered by her to the institute; and badges were awarded to the following nurses: Trained in Edinburgh—Mary Brown, Airdrie; Annie Clarke, Stirling; Mary Coats, Tarbert; Jane Douglas, Jedburgh; Alice Dewart, Islay; Jemima Elliot, Kilchoan; Emily French, Inverness; Elsie B. Low, Dumfries; Jessie Neilson, Elgin; Jessie C. Paul, Port Glasgow; Annie Telford, Ballantrae; Dora M. Walmsley, Kilmarnock; and Agnes B. White, Kirkcaldy. Trained in Glasgow—For Institute—Lizzie Carmichael, Susannah Collins, Agnes Kelly, and Julia Mackenzie. For Glasgow Staff—Bessie Haslam, Jane Mackenzie. Trained in Aberdeen—Jessie A. G. Clarke. Trained in Dundee—Annie G. Spence. Certificates were also presented to 21 nurses who had completed two years' service in connection with the institute.

The Marquis of Lorne briefly addressed the assembly, congratulating the nurses on still being in the same abode, and upon the success which had crowned their work. He hoped that the recent bazaar in Glasgow for the nurses of Argyllshire would prove a profitable example to other counties. He explained some remarks made in his speech last year with reference to sending money from Scotland to London, which, he thought, had been misinterpreted elsewhere. The practice pursued by the British Committee he considered an excellent one, for they had not only given back the money sent up by Scotland, but they had returned it with interest.

The Princess then visited Miss Buchanan, one of the nurses, who is dangerously ill. She had been on duty at Ardrishaig, where she narrowly escaped drowning, having been rescued by Miss Mary Weir, her landlord's daughter, whose efforts on this occasion have since been recognised by the Royal Humane Society.

### Where to Go.

The Portman Rooms, Baker Street, W.—A musical afternoon will be given on Thursday, December 15th, 1898, by Mrs. Edith A. Dick and Miss Dancer. Tickets (in advance) 3s. 6d. and (on the day) 5s. each, from all the usual agents. Mrs. St. Hill is the palmist, Mr. Alfred Capper the thought-reader, and amongst the musicians is Mr. Charles Capper, of whistling celebrity.

Dowdeswell Galleries.—An interesting collection of original sketches and photographs taken by Mr. Henry Savage Landor during his remarkable journey in the "Forbidden Land" will be on view at 160, Bond Street, on Monday, December 12th, and afterwards.

Sir Squire Bancroft will read his arrangement of Dickens' "Christmas Carol" at St. Martin's Town Hall, Charing Cross Road, on Thursday afternoon, January 12th, on behalf of the Royal London Ophthalmic Hospital, Moorfields.

Page(s) missing

### Everybody's Opinion.

[Correspondence on all subjects is invited, but we cannot in any way be responsible for the opinions expressed by our correspondents. No communication can be entertained if the name and address of the correspondent is not given, as a guarantee of good faith but not necessarily for publication, or unless one side of the paper only is written on.]

### NURSES AND POISONS.

"A FELLOW WORKER" writes: It was with sorrow I read of a nurse's fatal mistake in giving half an ounce of opium, and with surprise that among the comments the first thought which struck me was so entirely overlooked by others. Had the doctor ordered 30 minims of opium instead of half a dram the mistake might never have occurred. I have had many years' experience as nurse and matron, and have always, I think, seen the strong tinctures prescribed by minims. I consider half a dram has a random sound about it as applied to opium. Secondly, I do not think a nurse should be left in charge of opium and other night draughts until she can read fluently and write prescriptions.

### DOCTORS V. MIDWIVES.

ELEANOR HEATLEY, matron, St. Clement's Maternity, Fulham Palace Road, writes: I have noticed your articles in THE HOSPITAL of November 12th and 19th, and the letter of "Tory" and Miss Hannen's reply in last week's issue, and see that my home and name are mentioned therein. I beg to state most emphatically that I have not any arrangement whatever with any doctor to attend my abnormal cases of labour. The patient can herself send for any doctor she may wish to attend her, and she is responsible for his fee. As a matter of fact, one doctor is generally sent for as he is lecturer to my pupils, and has had great experience in maternity work. He is also the regular doctor for most of my patients and is nearest to my home; but I have sent to several others when I have wanted assistance, and I have never had any refuse to come to me, and have always found them pleased to have our assistance. I quite agree with Miss Hannen "that no self-respecting doctor would work under a midwife."

### HOSPITAL HOUSEKEEPING.

"ANOTHER MATRON" writes: The reproach of indifference in such an important matter as hospital housekeeping encourages me, though but a novice, to put down on paper a few thoughts that occurred to me in reading those interesting articles on "Hospital Housekeeping" by "A Matron." I have been on the nursing staff of four hospitals, and I say without hesitation the food was indifferent in all and positively objectionable in two of them—to such an extent, indeed, that through my complaints the attention of two committees was drawn to the subject. As sister in a large provincial hospital I complained over and over again of the inferiority of the food often sent up to the patients, and in this instance spoke to one of the hospital visitors of the unbroken monotony of rice pudding to which the patients were condemned; there was in future an alternative sago pudding provided about three times a week. Another smaller hospital containing over 100 beds provided me with the experience of cold ham for breakfast four, if not five, mornings out of seven (I do not mean to imply that this was the routine), a stinted butter allowance, and meals most unappetisingly served. Fresh from these memories I became matron, and I determined to arrange for a variety in diet both for patients and nurses, and I am met by three difficulties: First, the urgent necessity for economy; second, the matter of contracts with different tradespeople; and, lastly, by the limited time at the disposal of a matron for housekeeping. In the above-mentioned articles it was assumed that the housekeeper could herself inspect what was plentiful and cheap in the market on any given day, but is this a practicable theory? None of the matrons under whom I have worked could possibly have given so much time for this purpose. In my hospital it is altogether out of the question; other duties tie me indoors. The alternative is a call for orders; and by hospital custom a contract with a few tradesmen forms the limit of one's market; and though cabbages in the street or at the greengrocer's shop round the corner may be selling for 6d. a dozen, one must, by the exigencies of the case, buy twelve for 1s. from Messrs. Brown and Smith, and at longer intervals. One does not wish to dispute some advantages from the system of contracts, and for institutional accounts such an arrangement one imagines must be far simpler. But cheap marketing, in my opinion, must be done for ready money, and ready money is often at a premium in hospitals as elsewhere. But the question of variety in diet, happily, does not depend entirely on ready money or unlimited time for housekeeping. I can give my patients and nurses cod, whiting, plaice, fresh haddock, fresh herrings, hake, and an almost equal variety in meats and puddings, without being accused of extravagance—one of the matron's nightmares. My cases often require milk puddings, but baked apples, stewed quinces, and other fruits, even in small quantity, take off the monotony of ground rice, sago, semolina, rice, cornflour shape, &c. I find, too, suet pudding, baked and boiled bread puddings, and even occasional pastry have not retarded convalescence. We sadly need to forget many old traditions in hospital diet. One point in which I am distinctly conscious of failure is the breakfast meal. Except by special order from the doctor no patient in any hospital in which I have worked has ever been provided with anything but bread and butter and tea, and occasionally cocoa, for breakfast; and, knowing that this institution was to go on the lines of other hospitals, I had no alternative but to keep to those lines. Bacon, eggs, and fish, I do give on Sunday mornings to my patients, but if nurses and people in health require these every day why not convalescing patients? Ah! but the cost per head in the accounts would be sensibly increased if this very desirable addition to patients' breakfast could be brought about. I remember once asking a "visitor" if he could manage to get the sisters and nurses an allowance of jam once or twice a week for tea, and even an occasional cake, and he actually did follow up my remarks by appealing to the secretary on the subject, with the following result: That it was found it would cost 2d. a head per week extra to do this, and at the end of a year some hundreds of pounds. My request fell through. I am convinced, however, that it is cheaper and happier (I use the word advisedly) to provide nurses with thoroughly good and as much varied diet as possible. The food is eaten, and grumbling is reduced to a minimum.

### Nurses' Dances.

THE following letter appeared in the *Daily Mail* of November 21st:—

"'Nurses' dances' at public institutions is certainly a misnomer. These functions are organised solely for the pleasure and amusement of the higher placed officials, to which the nurses' recreation is subsidiary.

"For instance, at one of the Metropolitan Asylum Board's hospitals it is customary during the winter to allow the nurses, on two or three occasions, to remain up later than usual for the purpose of dancing, but they are not allowed to invite any outsiders. On the other hand, however, the medical superintendent, after dining a few male friends (not, I presume, at the ratepayers' expense), introduces such friends into the nurses recreation room, without so much as obtaining the nurses' permission, for the purpose of enabling them to spend the evening in flirting and dancing without the restrictions placed upon them in mixing with their social equals.

"That these 'gentlemen' are sometimes apt to forget what is due to them it is only necessary to mention that on one occasion last winter one of these friends of the medical superintendent at the hospital in question betrayed evidence of having dined too well, but his company was forced upon the nurses with no regard to their feelings. One can well understand that a nurse declining to dance with a friend (even though slightly elevated) of the medical superintendent would be likely to incur the displeasure of that official.

"With no desire to restrict the proper and sufficient recreation of the ratepayers' nurses or to raise the question of the propriety of 'sounds of revelry by night' amid the sick and dying, I suggest that on the occasion of these dances in the hospitals belonging to the ratepayers the nurses should have the privilege of inviting their own partners (of course limited to one each), and not be forced to accept the attentions of men who consider themselves the superiors in social importance of the nurses, and only look upon such nurses as providers of their evening's enjoyment.

"A NURSE'S BROTHER."

### For Reading to the Sick.

"SUBMISSION."

"NOT my will, but Thine be done."—*St. Luke* xxii. 42.

My God, my Father, while I stray,
Far from my home, in life's rough way,
O teach me from my heart to say—
"Thy will be done."

Though dark my path, and sad my lot,
Let me be still and murmur not,
Or breathe the prayer divinely taught—
"Thy will be done."

If Thou should'st call me to resign
What most I prize, it ne'er was mine;
I only yield Thee what is Thine—
"Thy will be done."

Let but my fainting heart be blest
With Thy sweet Spirit for its guest,
My God, to Thee I leave the rest—
"Thy will be done."

Renew my will from day to day,
Blend it with Thine, and take away
All that now makes it hard to say—
"Thy will be done."

Then when on earth I breathe no more
The prayer, oft mixed with tears before,
I'll sing upon a happier shore—
"Thy will be done."

Show me the path! I had forgotten Thee
When I was happy and free,
Walking down here in the gladsome light of the sun;
But now I come and mourn; oh set my feet
On the road to Thy blest seat!
And for the rest, oh God, Thy will be done!

—*J. Ingelow.*

God many a spiritual house has reared, but never one
Where lowliness was not laid first, the corner stone.

—*Trench.*

**Reading.**

"Not my will, but Thine be done."—*St. Luke* xxii. 42.

WISDOM: For how do we know what is food for us? If we were to have our own will and our own way mischief of all kinds might follow. No! the child must lean upon the Father's breast, and wait "while He walks." That is wisdom.

GRACE: Our Lord was "full of grace," and this is one of the trickling streams out of His fulness. Nature and grace speak different languages; the one earthly, the other heavenly. Nature says, "Not Thy will, but mine be done." Grace says, "Not my will, but Thine be done."

REST: It is when our wills are contrary to God's will—when He bids us go to Nineveh we will go to Tarshish—that the storm arises, and "the sea works and is tempestuous." When Jonahs are thrown overboard; when our wills are brought into conformity with God's will; when the same mind is in us "that was also in Christ Jesus"; then "the sea ceases from her raging," and there is a great calm.

COMFORT: For all is left with God; all cares are cast on Him; and we have simply to live like "the birds," and grow like "the lilies." Safe in Christ, and with no will of our own, there is comfort.—*Rev. Josiah Bateman.*

### Wants and Workers.

"MATRON OF AN ISOLATION HOSPITAL" begs to thank Miss Flint for kindly sending copies of "Misericordia." They will be most acceptable here.

### Notes and Queries.

**The contents of the Editor's Letter-box have now reached such unwieldy proportions that it has become necessary to establish a hard and fast rule regarding Answers to Correspondents. In future, all questions requiring replies will continue to be answered in this column without any fee. If an answer is required by letter, a fee of half-a-crown must be enclosed with the note containing the enquiry. We are always pleased to help our numerous correspondents to the fullest extent, and we can trust them to sympathise in the overwhelming amount of writing which makes the new rules a necessity.**

**Every communication must be accompanied by the writer's name and address, otherwise it will receive no attention.**

*L.O.S.*

(113) 1. Can I buy a L.O.S. certificate? I am a maternity nurse, and have nine years' experience. I have been coaching for the examination, but am frightened to go up for it.—*Nurse L.*

I am anxious to qualify for the L.O.S. examination. I cannot go to London before July, 1899, and wish to study privately in the meantime. 2. Will you give me particulars as to the date of examination, (3) books, &c.?—*Nurse M. F.*

I wish to go in for the L.O.S. examination. 2. How many cases must I have attended? Must I have a doctor's certificate? 3. What books should I study?—*Annette.*

1. The L.O.S. certificate cannot be gained except by passing an examination, of which the following are the regulations; 2. "Candidates must be 21 years of age, must produce certificates of moral character, proof of having attended a lying-in hospital or charity for not less than three months, of having personally attended not less than 25 labours, and of having attended a course of theoretical teaching by lectures or tutorial instruction satisfactory to the Board of Examiners." Written and oral examinations are held four times a year—in January, April, July, and October—at 20, Hanover Square, W., for which the fee is £1 1s., half of which is returned to unsuccessful candidates. 3. "A Practical Handbook of Midwifery," by Francis W. N. Haultain, M.D., price 6s. from the Scientific Press, can be recommended as useful.

*Massage versus Wardwork.*

(114) Will you kindly inform me if it requires greater strength of constitution to do about five or six hours massage a day than to do the average ward work of a general hospital? This supposing the masseuse to be in good muscular condition to start with. 2. Also, what the usual fee is per hour for a visiting masseuse?—*Flynn.*

It is impossible to give the exact ratio of the difference of strength required in general nursing and massage. All that can be said is that massage is much more exhausting. 2. From 5s. an hour.

*Army Nursing Service Reserve.*

(115) Would you kindly tell me for one of my probationers where to get information about the "Army Reserve Nursing"? She is anxious to feel she may join that when her training is finished.—*M. B.*

See "Burdett's Official Nursing Directory" (5s., from Scientific Press, p. 269). The address of secretary is 18, Victoria Street, S.W.

*Work for the Middle-aged.*

(116) Would you kindly inform me if there is any part of hospital work which needs no training, and which a lady of 55 could fill?—*E. E. B.*

If domesticated and active it might just be possible to find employment in the store-room or linen closet. Advertise and see, but we cannot feel hopeful of your success.

*Zenana Work.*

(117) Will you kindly give me the name of the association in London with which Lady Kinnaird is connected?—*Sister Margaret.*

Perhaps you mean the Zenana Bible and Medical Mission, 2, Adelphi Terrace, London, W.C., of which Lord Kinnaird is hon. treasurer.

*Visiting Nurses' Fees.*

(118) Kindly tell me what are the usual fees for a visiting nurse.—*Constant Reader.*

It varies according to the district and class of patient, from 1s. 6d. a visit upward.

*Non paying Probationer.*

(119) What hospital would take me without paying a premium? My age is 20. I am a Roman Catholic.—*M. A. S.*

Many hospitals receive probationers without premiums. See list of Children's Hospitals in Burdett's "Official Nursing Directory," published by the Scientific Press, Southampton Street, Strand, London, W.C.

*Domestic Probationer.*

(120) 1. Would having been in domestic service disqualify me for entering into hospital training? 2. Is it absolutely necessary I should know languages?—*H. B.*

There is a good demand for probationers who have been servants. 2. A fair elementary education only is necessary. Apply to the Matrons of the hospitals in your own town.

*Meat Extracts.*

(121) The recent disclosures concerning the horrible materials used in the manufacture of meat extracts have so shaken my confidence in their value that I should be very thankful if you could give the names of some makers, or extracts which one could rely on. I have often given Vimbos to some of the poor patients in connection with the medical mission I work with, as I thought it was easily prepared and nourishing.—*M. H. H.*

Amongst the many excellent preparations, we have had excellent analyses of Lipton's and Vimbos.

